# Chronic intermittent hypoxia induces the pyroptosis of renal tubular epithelial cells by activating the NLRP3 inflammasome

**DOI:** 10.1080/21655979.2022.2047394

**Published:** 2022-03-09

**Authors:** Chunyan Bai, Yingfei Zhu, Qiaoliang Dong, Yuwei Zhang

**Affiliations:** a Division of Geriatrics, Xiangya Second Hospital of Central South University, Changsha City, Hunan Province, China; b Division of International Medical Services, Xiangya Second Hospital of Central South University, Changsha City, Hunan Province, China

**Keywords:** Chronic intermittent hypoxia, pyroptosis, obstructive sleep apnea syndrome, NLRP3 inflammasome

## Abstract

Obstructive sleep apnea syndrome (OSAS) is a respiratory disorder and chronic intermittent hypoxia (CIH) is an important pathological characteristic of OSAS. Injuries on renal tubular epithelial cells were observed under the condition of CIH. Pyroptosis is a programmed mode of cell death following cell apoptosis and cell necrosis, which is mediated by NLRP3 signaling. The present study aims to investigate the effects of CIH on the pyroptosis of renal tubular epithelial cells and the underlying mechanism. Firstly, CIH was induced in two renal tubular epithelial cell lines, HK-2 cells and TCMK-1 cells. As the aggravation of hypoxia, an increasing trend of elevated apoptotic rate was observed in HK-2 cells and TCMK-1 cells, accompanied by the excessive release of ROS and LDH, and upregulation of NLRP3. Subsequently, the CIH model was established on rats. The pathological analysis results indicated that in CIH rats, the glomerular bottom membrane and mesangium were slightly thickened and edema was observed in the renal tubule epithelium. More serious injury was observed in the moderate intermittent hypoxia group. The expression level of IL-1β and IL-18 was promoted as the aggravation of hypoxia, accompanied by the elevated production of LDH and ROS. The expression level of cleaved Caspase-1, Caspase-1, GSDMD, TLR4, MyD88, NF-κB, p-NF-κB, and NLRP3 was found significantly upregulated as the aggravation of hypoxia. Lastly, the pathological changes in rats induced by CIH were dramatically abolished by MCC950, a specific inhibitor of NLRP3. Collectively, CIH triggered the pyroptosis of renal tubular epithelial cells by activating the NLRP3 inflammasome.

## GRAPHICAL ABSTRACT

**Figure:**
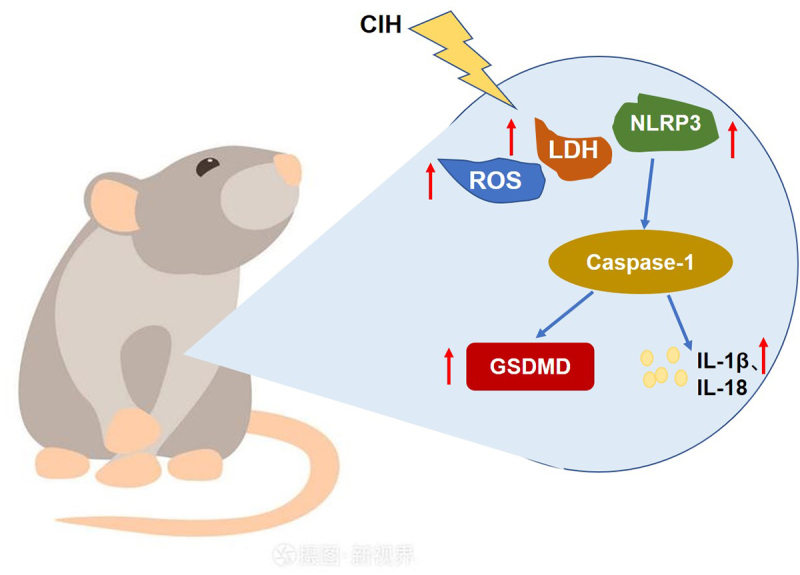

## Introduction

Obstructive sleep apnea syndrome (OSAS) is a respiratory disorder characterized by partial or complete upper airway obstruction during sleep and CIH is the main pathological characteristic of OSAS [1]. The injury on renal tubular epithelial cells is regarded as the main inducer in the progression of chronic kidney disease [2]. Normal renal tubular epithelial cells are structurally closely linked to the renal interstitial tight junction with strong metabolic activity and promising proliferation [3]. The interstitial inflammation and fibrosis will be directly triggered by the structural or functional damages on renal tubular epithelial cells, which further facilitates the infiltration of interstitial inflammatory cells and the proliferation of intrinsic cells, contributing to the development and processing of renal interstitial fibrosis [4].

Pyroptosis is a programmed and inflammatory mode of cell death following cell apoptosis and cell necrosis [5], which is mediated by 2 main pathways, including classical caspase-1 and non-caspase-1 pathways. Similar to apoptotic cells, nuclear shrinkage, exocytic vesicles, DNA breakage, positive TUNEL staining, and positive AnnexinV staining is also observed in pyroptosis cells. In addition, similar to necrotic cells, swelling, bursting, content (such as LDH) releasing, and inflammatory reactions are characteristics of pyroptosis cells [6]. Therefore, pyroptosis is also named cell inflammatory necrosis, which is reported to play an important role in multiple types of diseases, such as infection diseases [7], immune disease [8], and cardiovascular diseases [9].

The inflammasome is a protein complex formed by the assembly of a variety of proteins including pattern recognition receptor (PRR) and plays a critical role in stimulating and regulating renal inflammatory response, which mediates aseptic inflammation induced by tissue injury and is regarded as a receptor and regulator of inflammatory responses [10]. As an important family member of the inflammasome, nucleotide-binding oligomeric domain-like receptor protein 3 (NLRP3) inflammasome induces the excessive production of IL-1β and IL-18 by activating caspase-1 [11].

We suspected that CIH induced pathological changes are associated with the pyroptosis in renal tubular epithelial cells and the present study aims to investigate the possible pathological mechanism underlying the pyroptosis in CIH induced renal tubular epithelial cells by exploring the function of the NLRP3 inflammasome, which will provide a potential molecular target for the diagnosis and treatment of clinical OSAS.

## Materials and methods

**Cells, treatments, and grouping**: Human renal tubular epithelial cells, HK-2 cells, were purchased from Procell (CL-0109, Wuhan, China) and mouse renal tubular epithelial cells, TCMK-1 cells were obtained from Shanghai Zeye Biotechnology (Shanghai, China). Cells were cultured in DMEM/F12 medium containing 10% FBS under the condition of 5% CO_2_ and 37°C. After the cell density reached 60%-70%, cells were divided into 4 groups: Control, Mild-CIH, Moderate-CIH, and CH. In the control group, cells were treated with the blank medium. In the Mild-CIH group, cells were treated with hypoxia (5% O_2_) for 15 hours followed by reoxygenation for 9 hours with 2 circles (total for 48 hours). Cells in the Moderate-CIH group were treated with hypoxia (1% O_2_) for 15 hours followed by reoxygenation for 9 hours with 2 circles (total for 48 hours). In the CH group, cells were treated with hypoxia (1% O_2_) for a continuous 48 hours.

**Flow cytometry for the analysis of apoptosis** [12]: Cells were seeded on 6-well plates and incubated at 37°C for 48 h, followed by centrifugation at 300 g for 5 min. Cells were collected and resuspended with serum-free medium and approximately 5 μL Annexin V-FITC reagents (MULTI SCIENCES, Beijing, China) and 10 μL PI reagents (MULTI SCIENCES, Beijing, China), followed by incubation for 10 min at room temperature in the dark. Then, the cell suspension was mixed with PBS buffer in the flow tube and tested on NovoCyte™ (NovoCyte 2060 R, ACEA Biosciences, Hangzhou, China) for the analysis of apoptosis.

**CIH modeling** [13]: Twenty-three male Wistar rats (7–9 weeks) were purchased from Topgene Biological (Changsha, China) and were placed in the intermittent hypoxic chamber from 10:00 AM to 5:00 PM every day. Nitrogen and air were circulated into the intermittent hypoxic chamber for 8 min each time. The oxygen concentration in the intermittent hypoxic chamber was monitored by the oxygen control instrument to program the gas delivery and exhaust device so that the lowest oxygen concentration in the intermittent hypoxic chamber reached 8%-12% in each cycle. The oxygen concentration gradually returned to 21% as the low-oxygen gases were removed and the air was inhaled. The intervention lasted for 8 weeks.

Three groups were divided to determine the pathological state of CIH modeling and the activity of NLRP3 signaling: Control, Mild-CIH, and Moderate CIH group. Animals in the control group were treated with normal air and in the Mild-CIH group were treated with the intermittent hypoxic intervention with a minimum of 12% oxygen concentration. Rats in the Moderate CIH group were treated with intermittent hypoxic intervention with a minimum of 8% oxygen concentration.

Three groups were divided to check the function of NLRP3 signaling in the development of CIH: Control, Moderate CIH, and Moderate CIH+ Moderate-CIH+MCC950 groups. Animals in the control group were treated with normal air and in the Moderate CIH group were treated with the intermittent hypoxic intervention with a minimum of 8% oxygen concentration. Rats in the Moderate-CIH+MCC950 group were treated with the intermittent hypoxic intervention with a minimum 8% of oxygen concentration, followed by intraperitoneal injection with 10 mg/kg/day MCC950 for 14 days.

Animals were sacrificed by intraperitoneal injection with 3% 50 mg/kg pentobarbital sodium at the end of the experiment.

**Real-time PCR** [14]: After isolating total RNAs from cells and tissues using the TRIzol reagent (CW0580S, CWBIO, Beijing, China), cDNA was obtained by the transcription from 2 µg sample of RNA with a HiScript II Q RT SuperMix Kit (R223-01, Vazyme, Nanjing, China). The RT-PCR was conducted with a 7500 Real-Time PCR System (ABI, California, USA) using the SYBR Green PCR Master Mix (A4004M, Lifeint, Xiamen, China). Lastly, the gene expressions were calculated utilizing the 2^−ΔΔCt^ method following being normalized with β-actin. The sequences of primers were shown in Table 1.

**Table 1.** The sequences of the primers

Genes	Sequences (5’-3’)
β-actin F	GCCATGTACGTAGCCATCCA
β-actin R	GAACCGCTCATTGCCGATAG
IL-1βF	CAGACCCCAAAAGATTAAGGATTG
IL-1βR	CTAGCAGGTCGTCATCATCC
IL-18 F	GGAATCAGACCACTTTGGCA
IL-18 R	GGGATTCGTTGGCTGTTCG

**Western blotting assay** [15]: After isolating total proteins from HESCs or nucleus with the lysis buffer, a BCA kit (Abcam, Cambridge, UK) was utilized to determine the concentration of proteins, followed by loading the proteins into the 12% SDS-PAGE. After separation, proteins in the gel were transferred onto the PVDF membrane (IPVH00010, Massachusetts, USA), followed by incubation in the TBST buffer containing the primary antibody against Cleaved Caspase-1 (1:1000, 4199 T, CST, Massachusetts, USA), p-NF-κB (1:1000, 3033, CST, Massachusetts, USA), Caspase-1 (1:1000, MA5-32,909, ThermoFisher, Massachusetts, USA), GSDMD (1:500, af4013, Affinity, California, USA), TLR4 (1:500, 19,811-1-ap, proteintech, Chicago, USA), GSDMD (1:500, af5195, Affinity, California, USA), NF-κB (1:500, 66,535-1-lg, proteintech, Chicago, USA), NLRP3 (1:500, ab263899, Abcam, Cambridge, UK), and β-actin (1: 2000, TA-09, ZSGB-BIO, Beijing, China). After incubating at 4°C overnight, the membrane was incubated with the secondary antibody (1:2000, ZB-2305, ZSGB-BIO, Beijing, China) at room temperature for 90 min. Lastly, the ECL solution was utilized to expose the membrane and the expression of proteins was quantified using the Image J software.

**ELISA assay** [16]: The release of LDH and ROS was determined by ELISA using the commercial kits (MEIMIAN, Beijing, China) according to the instruction of the manufacturer. In brief, the supernatants or homogenates were collected and planted in the 96-well plate along with the 5 gradient concentrations of standards. After being incubated for 90 min at room temperature, the conjugate reagents were added to be incubated for 90 min at room temperature, followed by adding the TMB solution for 15 min. Lastly, the stop solution was added and the microplate reader (WD-2102B, LiuYi, Beijing, China) was utilized to measure the absorbance at 450 nm.

**HE staining** [17]: Following washing renal tissues several times, tissues were dehydrated using different concentrations of ethyl alcohol solution, followed by transparentized with xylene for 15 min. The paraffin was used to embed tissues for 60 min, followed by cutting into slides. After baking, dewaxing, and hydration, slides were stained with hematoxylin solution for 3 min, followed by incubated with the hydrochloric acid for 15s and 3 washes. Slides were stained with eosin solution for 3 min, followed by washes, dehydration, transparentizing, and sealing. Lastly, images were taken under the inverted microscope (BX43, Olympus, Tokyo, China).

**Masson staining** [18]: The deparaffined sections were incubated with the Bouin solution at 37°C for 2 hours, followed by 3 washes and staining with celestine blue dye for 3 min. After being stained with Mayer hematoxylin for 3 mins, cells were differentiated with the acidic ethanol differentiation solution for seconds, followed by washing for 10 min. Slides were then stained with ponceau solution for 10 min, followed by being treated with phosphomolybdic acid for 10 min. The aniline blue dyeing solution was utilized to stain the slides for 5 min, followed by dehydration with 95% ethyl alcohol and absolute ethyl alcohol successively. Lastly, the images were taken under the inverted microscope (BX43, Olympus, Tokyo, China).

**PAS staining** [18]: The deparaffined sections were incubated with the oxidants for 15–20 min at room temperature, followed by being washed twice. Then, the Schiff Reagent was added and incubated in the dark for 10–20 min, followed by being washed by the sodium sulfite solution twice. Lastly, slides were washed and redyed with the Mayer hematoxylin for 2 mins, followed by taking images using the inverted microscope (BX43, Olympus, Tokyo, China).

**TUNEL staining** [19]: Paraffin slices were roasted and then dewaxed and hydrated. The sections were transferred to a wet box, and 50 μg/ mL Proteinase K working solution was added to each sample, and the reaction time was 30 min at 37°C. After washing with PBS 3 times, a sufficient amount of TUNEL detection solution was added to each sample and the samples were incubated at 45°C for 2 h in the dark. Lastly, the film was sealed for microscopic examination.

**Statistical analysis**: Data were expressed as mean ± SD and the Graphpad software was utilized for the analysis of data. The Student’s t-test was utilized to determine the difference between the 2 groups and the one-way ANOVA method was used to analyze the difference among more than 2 groups. p < 0.05 was taken as a significant difference.

## Ethics statement

We declare that all animal experiments involved in this manuscript were authorized by the ethical committee of The Second Xiangya Hospital, Central South University (Approval No. 2,021,009) and performed according to the guidelines for care and use of laboratory animals and the principles of laboratory animal care and protection. The document of ethical approval has been provided as ‘Supplementary document 1’.

## Results

We suspected that CIH induces significant pathological changes in rats by triggering pyroptosis in renal tubular epithelial cells. The present study aims to investigate the possible pathological mechanism underlying the pyroptosis in CIH-induced renal tubular epithelial cells by exploring the function of the NLRP3 inflammasome. We first established the in vitro CIH model in both HK-2 cells and TCMK-1 cells, followed by evaluating the apoptosis, detecting the release of ROS and LDH, and determining the expression level of NLRP3. The CIH model was then established in rats, followed by evaluating the pathological changes and apoptotic state in renal tissues. The expression level of Caspase-1, cleaved-caspase-1, the activity of GSDMD pathway, and the release of IL-1β and IL-18 were subsequently determined. Lastly, the involvement of NLRP3 in CIH-induced pathological changes in rats was verified by introducing MCC950, an NLRP3 specific inhibitor.

**Figure 1.**
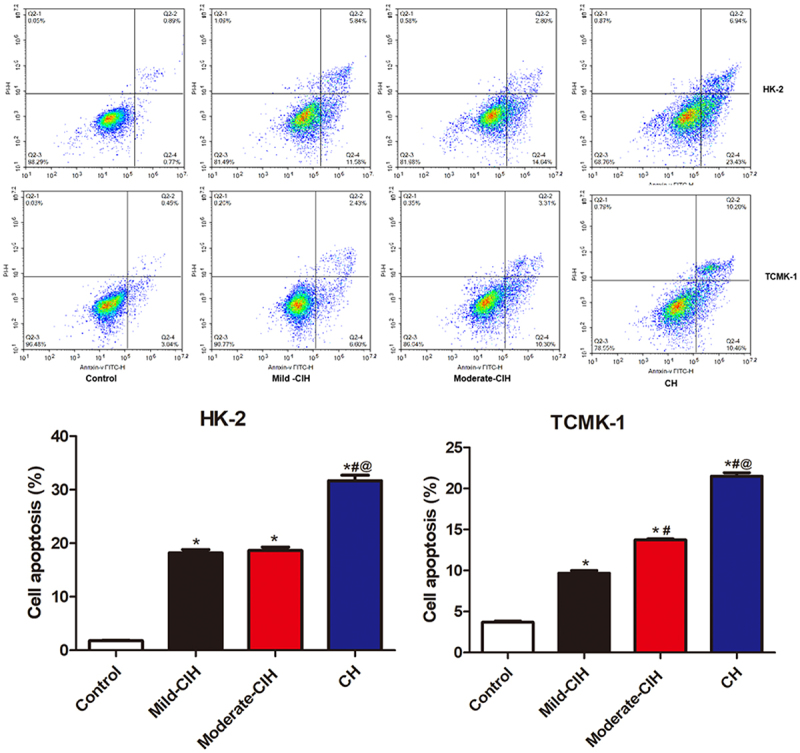
The apoptosis state of HK-2 cells and TCMK-1 cells was evaluated by flow cytometry after being treated with different strategies of CIH (n = 3, *p < 0.05 vs. control, #p < 0.05 vs. Mild-CIH, @ p < 0.05 vs. Moderate-CIH).

**CIH aggravated the apoptosis in renal tubular epithelial cells**: Flow cytometry was utilized to evaluate the state of apoptosis in renal tubular epithelial cells following different strategies of hypoxia. As shown in Figure 1, in both HK-2 cells and TCMK-1 cells, compared to control, the apoptotic rate was significantly elevated in the Mild-CIH, Moderate-CIH, and CH groups. In addition, as the aggravation of hypoxia, an elevated trend of increasing apoptotic rate was observed in HK-2 cells and TCMK-1 cells (*p < 0.05 vs. control, #p < 0.05 vs. Mild-CIH, @ p < 0.05 vs. Moderate-CIH).

**Figure 2.**
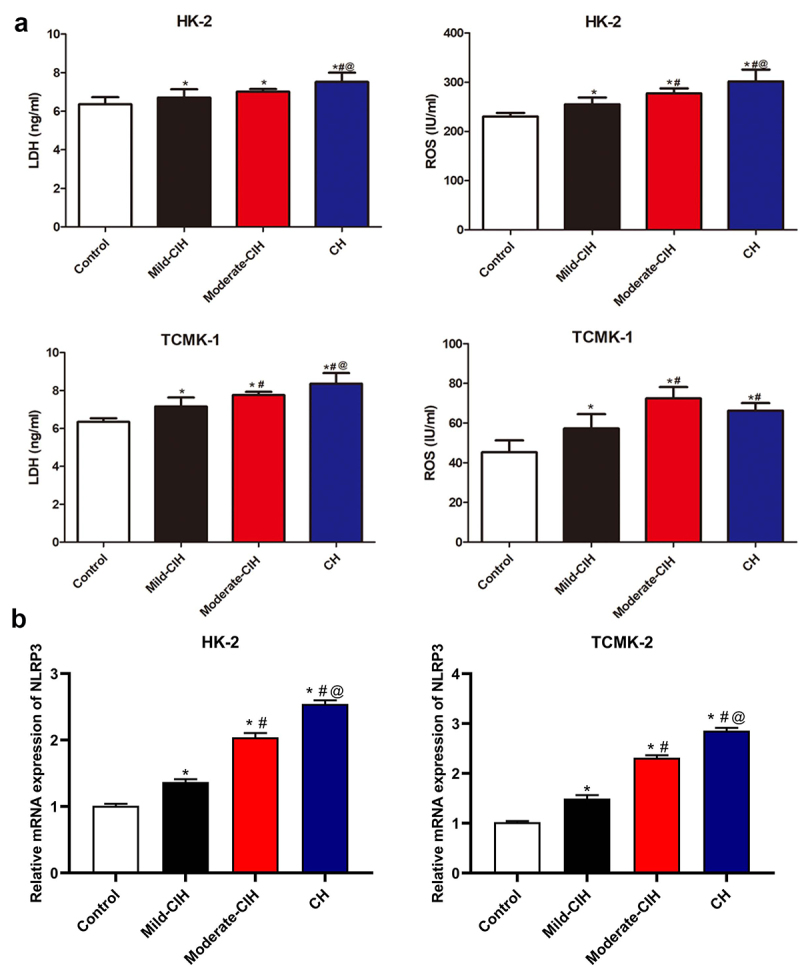
A. The release of LDH and ROS in HK-2 cells and TCMK-1 cells was measured by ELISA after being treated with different strategies of CIH. B. The relative gene expression level of NLRP3 in HK-2 cells and TCMK-1 cells was determined by RT-PCR assay (n = 3, *p < 0.05 vs. control, #p < 0.05 vs. Mild-CIH, @ p < 0.05 vs. Moderate-CIH).

**CIH facilitated the production of LDH and ROS, and increased the expression level of NLRP3 in renal tubular epithelial cells**: To evaluate the effects of CIH on the pathological state of renal tubular epithelial cells, ELISA was conducted to measure the release of LDH and ROS following different strategies of hypoxia. As shown in Figure 2(a), in HK-2 cells and TCMK-1 cells, compared to control, the concentration of LDH and ROS was dramatically elevated in the Mild-CIH, Moderate-CIH, and CH groups. Additionally, in HK-2 cells, the release of LDH and ROS increased as the aggravation of hypoxia. In TCMK-1 cells, the production of LDH was promoted as the aggravation of hypoxia. Although a slightly declined release of ROS was observed in the CH group compared to Moderate-CIH, compared to the Moderate-CIH group, the concentration of ROS was significantly elevated in the Moderate-CIH group (*p < 0.05 vs. control, #p < 0.05 vs. Mild-CIH, @ p < 0.05 vs. Moderate-CIH).

Furthermore, we investigated the expression level of NLRP3 in different groups. As shown in Figure 2(b), in both HK-2 cells and TCMK-1 cells, compared to control, NLRP3 was significantly upregulated as the aggravation of hypoxia (*p < 0.05 vs. control, #p < 0.05 vs. Mild-CIH, @ p < 0.05 vs. Moderate-CIH).

**Figure 3.**
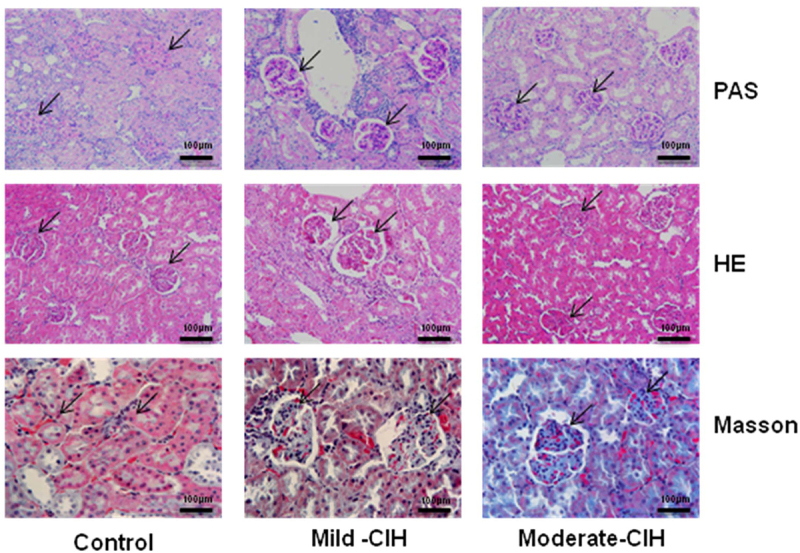
The pathological changes in renal tissues of rats were evaluated by PAS, HE, and Masson staining following different strategies of CIH (n = 3, *p < 0.05 vs. control, #p < 0.05 vs. Mild-CIH).

**CIH induced significant pathological changes in renal tissues of rats**: We further established the CIH model in rats, followed by evaluating the pathological changes in renal tissues using the PAS, HE, and Masson staining assays. As shown in Figure 3, in the control group, the structure of the glomerular and tubular epithelium was neat, without obvious thickening of the glomerular basement membrane and mesangium. The brush border of renal tubular epithelial cells was intact. In the Mild-CIH group, mild thicken glomerular basement membrane and mesangium was observed, with patchy brush border of renal tubular epithelial cells, mild hyperplasia in the mesangium, and mild infiltration of inflammatory cells. Severer pathological changes were observed in the Moderate-CIH group, with the glomerulus filling the entire renal vesicle and the significant infiltration of inflammatory cells.

**Figure 4.**
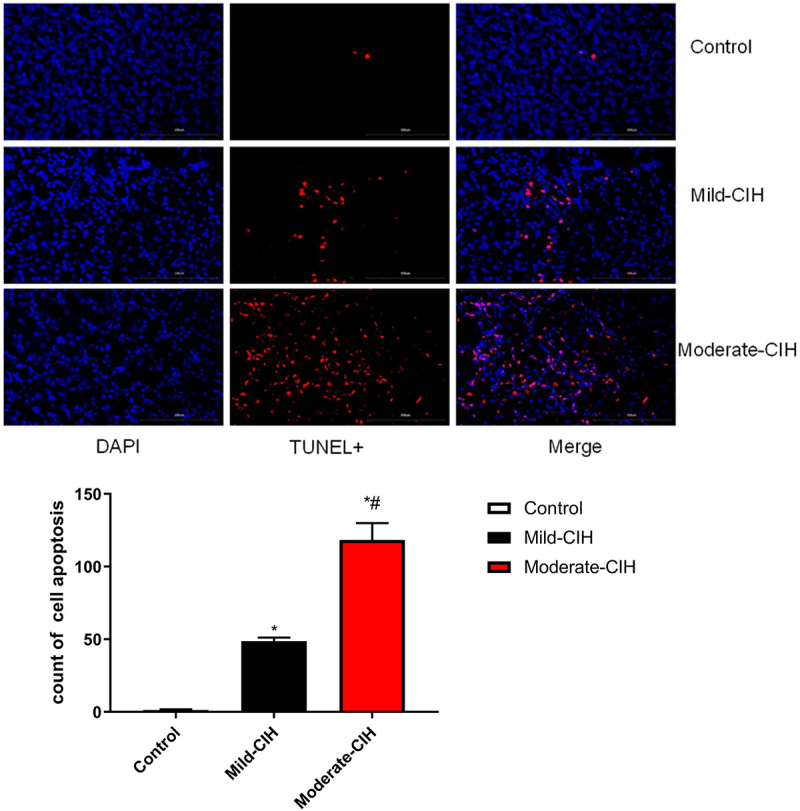
The apoptosis in renal tissues of rats was determined by the TUNEL staining assay (n = 3, *p < 0.05 vs. control, #p < 0.05 vs. Mild-CIH, @ p < 0.05 vs. Moderate-CIH).

**CIH induced dramatical apoptosis in renal tissues of rats**: As shown in Figure 4, TUNEL staining results showed that the apoptosis was aggravated with the increase of the degree of hypoxia. Compared to the control group, the apoptosis in the Mild-CIH and Moderate-CIH group was significantly increased (*p < 0.05 vs. control, #p < 0.05 vs. Mild-CIH).

**Figure 5.**
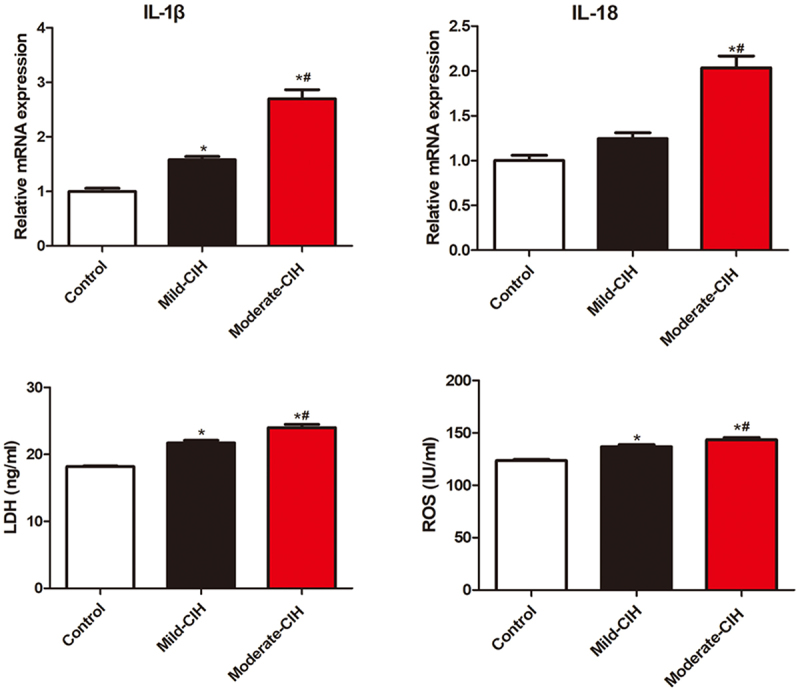
The production of IL-1β, IL-18, LDH, and ROS in renal tissues of rats was measured by ELISA following different strategies of CIH (n = 3, *p < 0.05 vs. control, #p < 0.05 vs. Mild-CIH).

**The elevated expression level of IL-1β, IL-18, LDH, and ROS was observed in CIH rats**: As shown in Figure 5, compared to control, significantly upregulated IL-1β, LDH, and ROS were observed in the Mild-CIH group and Moderate-CIH group. Compared to control, the expression level of IL-18 was dramatically promoted in the Moderate-CIH group. In addition, compared to the Mild-CIH group, the release of IL-1β, IL-18, LDH, and ROS was all elevated in the Moderate-CIH group (*p < 0.05 vs. control, #p < 0.05 vs. Mild-CIH).

**Figure 6.**
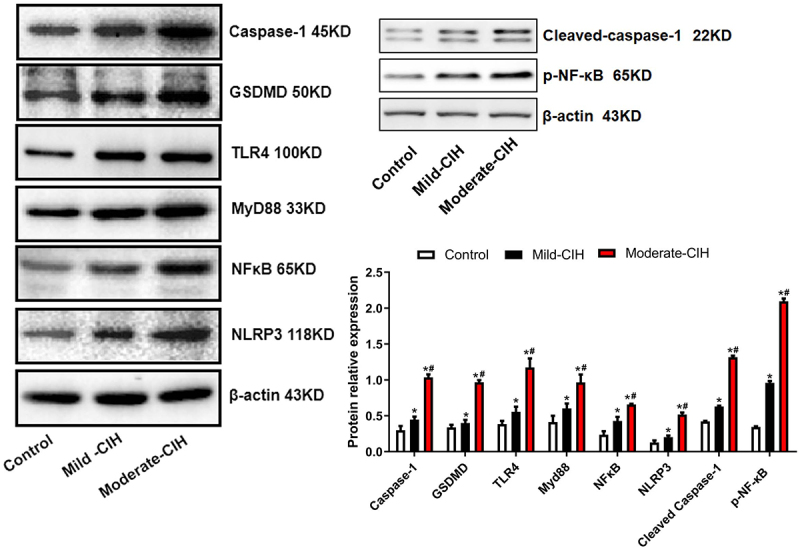
The expression level of Cleaved Caspase-1, Caspase-1, GSDMD, TLR4, MyD88, p-NF-κB, NF-κB, and NLRP3 in renal tissues of rats was measured by Western blotting assay following different strategies of CIH (n = 3, *p < 0.05 vs. control, #p < 0.05 vs. Mild-CIH).

**NLRP3 inflammasome pathway was significantly activated in CIH rats**: To explore the potential pathological mechanism of CIH on renal injury, the expression level of NLRP3 inflammasome related proteins was measured by Western blotting assay. As shown in Figure 6, compared to control, Caspase-1, Cleaved Caspase-1, GSDMD, TLR4, MyD88, p-NF-κB, NF-κB, and NLRP3 were found significantly upregulated in the Mild-CIH group and Moderate-CIH group. In addition, compared to the Mild-CIH group, the expression level of Caspase-1, Cleaved Caspase-1, GSDMD, TLR4, MyD88, p-NF-κB, NF-κB, and NLRP3 was greatly elevated in the Moderate-CIH group (*p < 0.05 vs. control, #p < 0.05 vs. Mild-CIH). These data revealed that CIH significantly induced the activation of the NLRP3 inflammasome pathway in renal tissues.

**Figure 7.**
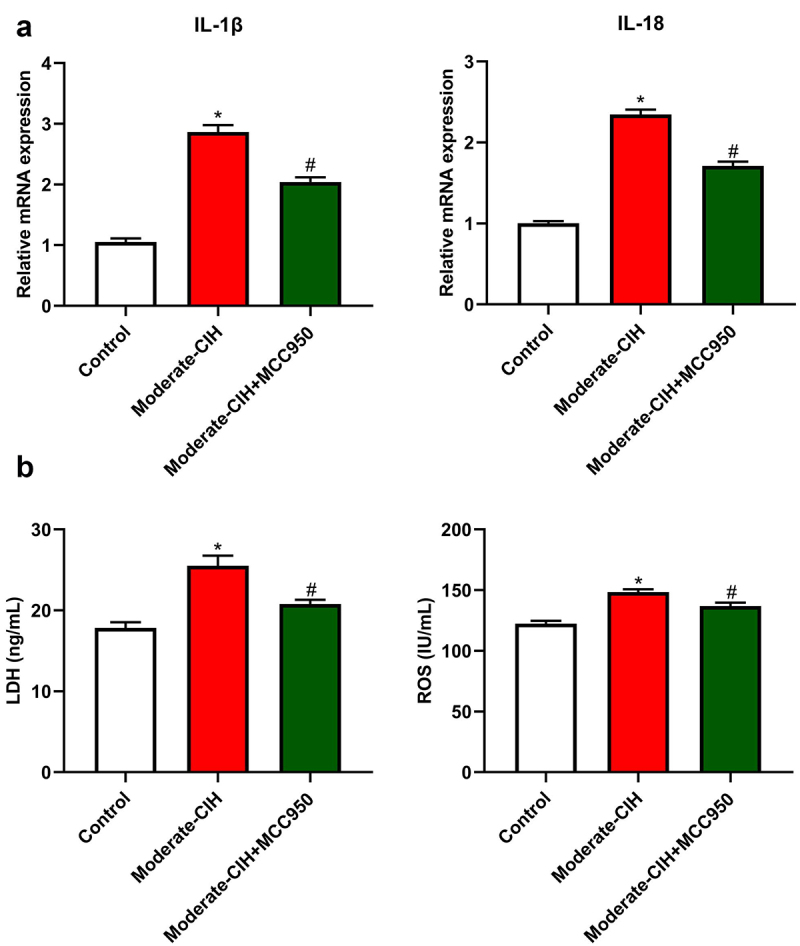
MCC950 reversed the effects of moderate CIH in rats. a. The relative expression level of IL-1β and IL-18 was determined by RT-PCR assay. b. The release of LDH and ROS was measured by ELISA. (n = 3, *p < 0.05 vs. control, #p < 0.05 vs. Moderate-CIH).

**NLRP3 inhibitor reversed pathological changes in the moderate CIH animals**: To evidence the involvement of the NLRP3 pathway in the development of CIH, MCC950, an NLRP3 inhibitor, was used to administer animals in the Moderate-CIH group. As shown in Figure 7, the elevated expression level of IL-1β and IL-18 in the Moderate-CIH group was dramatically repressed by the treatment of MCC950. Furthermore, the increased release of LDH and ROS in the Moderate-CIH group were significantly declined by the administration of MCC950 (*p < 0.05 vs. Control, #p < 0.05 vs. Moderate-CIH). These data confirmed that the NLRP3 pathway was involved in the development of CIH.

## Discussion

The pathological mechanism of systemic organ damage caused by OSAS mainly includes oxidative stress, systemic inflammatory response, hypoxia, and endothelial dysfunction. Several biological processes, such as increased apoptosis, excessive oxidative stress, marked inflammatory responses, and endothelial dysfunction, are reported to be associated with CIH [20]. In addition, activated oxidative stress, a key mechanism of endothelial dysfunction, is closely associated with the development of OSAS [21], which facilitates the inflammatory responses in blood vessels and kidneys. As the inducer and mediator of inflammatory responses and cytokines, oxidative stress plays a crucial role in the pathological process of diseases [22]. In the present study, intermittent hypoxia was applied on HK-2 cells, a human renal tubular epithelial cell line, and TCMK-1 cells, a mouse renal tubular epithelial cell line. We found that significant apoptosis was induced in renal tubular epithelial cells by the treatment of intermittent hypoxia.

Both oxidative stress and sympathetic stress are reported to be induced by CIH [23]. Under regular circumstances, a stable balance between the production and the elimination of ROS is maintained, which can be disrupted by CIH to induce severe oxidative stress [24]. Excessive production of ROS, as well as the accumulation of oxygen free radicals (superoxide anions, hydrogen peroxide, and hydroxyl radicals), will be induced under the state of oxidative stress, which further contributes to severe damages on key proteins and DNA in cells [25]. In addition, the denaturation of proteins, declined activity of the related ion-dependent adenosine triphosphate (ATP) enzyme in cardiomyocytes, and unstable activity of ATP enzyme on the cell membrane are reported to be induced by oxidative stress, which further results in the injuries on cardiomyocytes and the imbalance of the internal and external environment of the cell membrane, finally contributing to the rupture of the cell membrane. Under the disruption state of the cell membrane, LDH will be released and detected in the circulation [26,27]. In the present study, we found that the excessive release of ROS and LDH was significantly induced in both CIH treated renal tubular epithelial cells and CIH treated rats. It is reported that the permeability of the myocardial cell membrane is enlarged by the treatment of intermittent ischemia and hypoxia, which further leads to the death of myocardial cells and ventricular enlargement. Myocardial cells are subsequently stretched mechanically by the enlarged ventricle to induce the destruction of the myocardial cell membrane, which finally contributes to increased LDH release and elevated concentration of LDH in the circulation [28].

Under the stimulation of CIH, the systemic and local inflammatory responses will be induced by the excessive accumulation of ROS [29], which further results in the increased the production of pro-inflammatory cytokines, including IL-1β and 18 and exacerbates the inflammatory response [30]. It is reported that under the condition of CIH, significantly promoted production of pro-inflammatory cytokines is observed in the liver tissue of animals, accompanied by severe the inflammatory response and fibrosis in the liver lobules, suggesting that the liver tissue is one of the main organs that are severely impacted by CIH [31]. Previous research showed that [32] CIH induced production of IL-1β and ROS could be dramatically repressed by the treatment of taurodeoxycholic acid, which further verified that the tissue damage could be repaired by ameliorating the oxidative stress and inflammatory response. In the present study, the animal experiments revealed that the secretion of IL-1β and IL-18 was significantly elevated by the stimulation of CIH, indicating a severe state of inflammatory response induced by CIH.

Activation of the NLRP3 inflammasome signaling pathway is reported to promote the production of inflammatory factors and induce pyroptosis, resulting in severe inflammatory injury [33]. NLRP3 inflammasome functions primarily by activating caspase-1, shearing IL-1β and IL-18 precursors, and enabling the mature of IL-1β and IL-18 to exert the pro-inflammatory role. Dual signal stimulations are required for the activation of NLRP3 inflammasome [34]. Firstly, the pre-stimulation signal is mediated by pattern recognition receptors (PRRs), such as the toll-like receptor (TLR) family. After recognizing the specific ligands, TLRs upregulates the expression of NLRP3, Pro-IL-1, and Pro-IL-18 by activating the NF-κB pathway [35]. For example, LPS directly upregulates the expression of NLRP3 inflammasome in renal epithelial cells by binding with TLR4 [36]. In the secondary signal, pro-IL-1β and pro-IL-18, generating from the pre-stimulation signal, are sheared to produce the mature and active inflammatory factors [37,38]. In NLRP3 mediated pyroptosis, the activating of TLR4 induces the phosphorylation of I-κB through the MyD88 pathway, which further triggers the separation of NF-κB from I-κB and induces the transferring of NF-κB from the cytoplasm to the nucleus. As a consequence, the activation of NLRP3 inflammasome will be induced and the pro-inflammatory factors will be released, which finally contributes to the development of pyroptosis [39]. Additionally, the activation of NLRP3 inflammasome and secretion of IL-1β and IL-18 can be directly facilitated by the excessive produced ROS [40]. In the processing of inflammatory necrosis of cells, Gasdermin D (GSDMD) plays a critical role [41] and the inflammatory necrosis can be dismissed by downregulating GSDMD. In addition, GSDMD mediated pyroptosis is regarded as an important target for multiple inflammatory diseases [42]. In the present study, we found that the upregulation of Caspase-1, GSDMD, TLR4, MyD88, NF-κB, and NLRP3 in renal tissues could be induced by the stimulation of CIH, indicating that the pyroptosis in renal tubular epithelial cells could be induced by CIH by activating the NLRP3 inflammasome.

## Conclusion

Taken together, CIH induced the caspase-1 activation by upregulating the expression of LDH, ROS, and NLRP3. On the one hand, activated Caspase-1 cleaved GSDMD. On the other hand, activated Caspase-1 sheared pro-IL-1β and pro-IL-18 to release mature IL-1β and IL-18. Finally, pyroptosis in renal tubular epithelial cells was induced.

## Supplementary Material

Supplemental Material
