# Supplementary material for: Chronic intermittent hypoxia induces the pyroptosis of renal tubular epithelial cells by activating the NLRP3 inflammasome
Source: Bioengineered. 2022 Mar 9;13(3):7528–40. doi: 10.1080/21655979.2022.2047394 (PMC8973594; doi:10.1080/21655979.2022.2047394)
Supplement: Supplemental Material [file KBIE_A_2047394_SM8966.zip › supplementary/downloadFromZipFile.pdf]

中南大学湘雅二医院实验动物福利伦理审查同意书

Approval of Animal Ethic

The Second Xiangya Hospital, Central South University

批准编号 Approval No.	2021009

本动物实验方案经过中南大学湘雅二医院实验动物伦理委员会审核，符合动物保护、动物福利和伦理原则，符合国家实验动物福利伦理的相关规定。The animal use protocol listed below has been reviewed and approved by the Institutional Animal Care and Use Committee (IACUC), The Second Xiangya Hospital, Central South University, China

<table>
<tr><td rowspan="2">实验名称
Study Title</td><td colspan="6">慢性间歇性缺氧激活NLRP3炎性小体通路致肾脏小管上皮细胞焦亡的机制研究</td></tr>
<tr><td colspan="6">Mechanism of chronic intermittent hypoxia induced pyroptosis of renal tubular epithelium by NLRP3 inflammasome pathway</td></tr>
<tr><td rowspan="2">实验申请人
Applicant</td><td>张聿炜</td><td rowspan="2">职称/学位
Title/Degree</td><td>主治医师/博士</td><td rowspan="2">邮箱
Email</td><td rowspan="2" colspan="2">zhangyuwei5050@csu.edu.cn</td></tr>
<tr><td>Yuwei Zhang</td><td>Physician/Ph.D</td></tr>
<tr><td rowspan="2">项目负责人
Principle Investigator (PI)</td><td>张聿炜</td><td rowspan="2">职称/学位
Title/Degree</td><td>主治医师/博士</td><td rowspan="2">邮箱
Email</td><td rowspan="2" colspan="2">zhangyuwei5050@csu.edu.cn</td></tr>
<tr><td>Yuwei Zhang</td><td>Physician/Ph.D</td></tr>
<tr><td rowspan="2">院系(部门)
Department</td><td colspan="2">中南大学湘雅二医院国际医疗部</td><td colspan="2" rowspan="2">申请日期
Application date</td><td colspan="2" rowspan="2">2021年1月15日</td></tr>
<tr><td colspan="2">The International Medical Department of the Second Xiangya Hospital ，Central South University</td></tr>
<tr><td rowspan="2">动物种系
Species or Strains</td><td colspan="2">Wistar 大鼠</td><td colspan="2" rowspan="2">动物数量 Quantity</td><td colspan="2" rowspan="2">60只</td></tr>
<tr><td colspan="2">Wistar rats</td></tr>
<tr><td>计划执行时间
Period of experiments</td><td colspan="2">2021年2月1日至2022年12月31日</td><td colspan="2">实验动物使用许可证
License No. of Laboratory Animal Facility</td><td colspan="2">SYXK（湘）2017-0002</td></tr>
<tr><td>审查意见
Results from committee</td><td colspan="6">【 √ 】符合动物福利伦理要求，同意实验 Agree

【 】调整方案后，可进行实验 Agree after modification

【 】不同意 Disagree</td></tr>
</table>

中南大学湘雅二医院实验动物伦理委员会

Animal Ethical and Welfare Committee，The Second Xiangya Hospital, CSU，P.R.China

日期（Date）：2021.1.18

中南大学湘雅二医院 实验动物伦理委员会
