# Supplementary material for: Chronic intermittent hypoxia induces the pyroptosis of renal tubular epithelial cells by activating the NLRP3 inflammasome
Source: Bioengineered. 2022 Mar 9;13(3):7528–40. doi: 10.1080/21655979.2022.2047394 (PMC8973594; doi:10.1080/21655979.2022.2047394)
Supplement: Supplemental Material [file KBIE_A_2047394_SM8966.zip › supplementary/Original WB images with marks for distinguishment.doc]

1. **NLRP3 118KD**

118KD

118KD

118KD

**2.TLR4 100KD**

100KD

100KD

100KD

**3.NFκB 65KD**

65KD

65KD

65KD

4. **GSDMD 50KD**

50KD

50KD

50KD

**5. Caspase-1 45KD**

45KD

45KD

45KD

**6. β-actin 43KD**

45KD

45KD

45KD

**7. MyD88 33KD**

45KD

45KD

45KD
